# Formation of quantum dots in GaN/AlGaN FETs

**DOI:** 10.1038/s41598-020-72269-z

**Published:** 2020-09-22

**Authors:** Tomohiro Otsuka, Takaya Abe, Takahito Kitada, Norikazu Ito, Taketoshi Tanaka, Ken Nakahara

**Affiliations:** 1 Research Institute of Electrical Communication, Tohoku University, 2-1-1 Katahira, Aoba-ku, Sendai, 980-8577 Japan; 2 Center for Spintronics Research Network, Tohoku University, 2-1-1 Katahira, Aoba-ku, Sendai, 980-8577 Japan; 3 Center for Science and Innovation in Spintronics, Tohoku University, 2-1-1 Katahira, Aoba-ku, Sendai, 980-8577 Japan; 4 Center for Emergent Matter Science, RIKEN, 2-1 Hirosawa, Wako, Saitama, 351-0198 Japan; 5 ROHM Co., Ltd, 21 Saiinnmizosakicho, Ukyo-ku, Kyoto, 615-8585 Japan

**Keywords:** Nanoscience and technology, Condensed-matter physics

## Abstract

GaN and the heterostructures are attractive in condensed matter science and applications for electronic devices. We measure the electron transport in GaN/AlGaN field-effect transistors (FETs) at cryogenic temperature. We observe formation of quantum dots in the conduction channel near the depletion of the 2-dimensional electron gas (2DEG). Multiple quantum dots are formed in the disordered potential induced by impurities in the FET conduction channel. We also measure the gate insulator dependence of the transport properties. These results can be utilized for the development of quantum dot devices utilizing GaN/AlGaN heterostructures and evaluation of the impurities in GaN/AlGaN FET channels.

## Introduction

GaN and the heterostructures are attractive materials because of their interesting electronic properties: the large direct bandgap, the high electron densities and mobilities. They are utilized in light-emitting diodes^1–3^, power and high-frequency electronics devices^4–6^. In electronic device applications, GaN/AlGaN heterostructures are important structures. High density and high mobility 2DEG is formed at the interface^7,8^. The 2DEG is also investigated on the viewpoint of spin-orbit interactions^9–11^ and electron spin resonances^12^. Quantum nanostructures can be fabricated from the heterostructure by utilizing nano-fabrications. Quantum point contacts^13^ and single electron transistors^14^ are reported. GaN/AlGaN nanowires^15,16^ and self-assembled GaN islands^17^ are also used to form quantum dots. Then GaN and the heterostructures are attractive also in quantum devices utilizing the electronic properties.

Quantum dots can be formed also by intrinsic impurity potentials not only by the electric gates or edges defined structures. In Si FETs, the formation of quantum dots by electrical potentials induced by dopants is reported^18–20^. Dopants themselves work as quantum dots and control of the dopants^21^ is used for quantum bit applications^22–26^, which is studied for quantum information processing^27,28^. The stronger confinement by the dopant makes larger quantization energies and this enables high-temperature operation of the semiconductor quantum bits^29^.

In this paper, we measure electron transport through GaN/AlGaN FETs at cryogenic temperature. We observe non-monotonic modulation of the current indicating formation of quantum dots near the pinch-off condition of the FET channel. Multiple quantum dots are formed in the potential fluctuations induced by the impurities near the conduction channel. We also measure the gate insulator dependence.

## Results

### Device and FET properties

**Figure 1:**
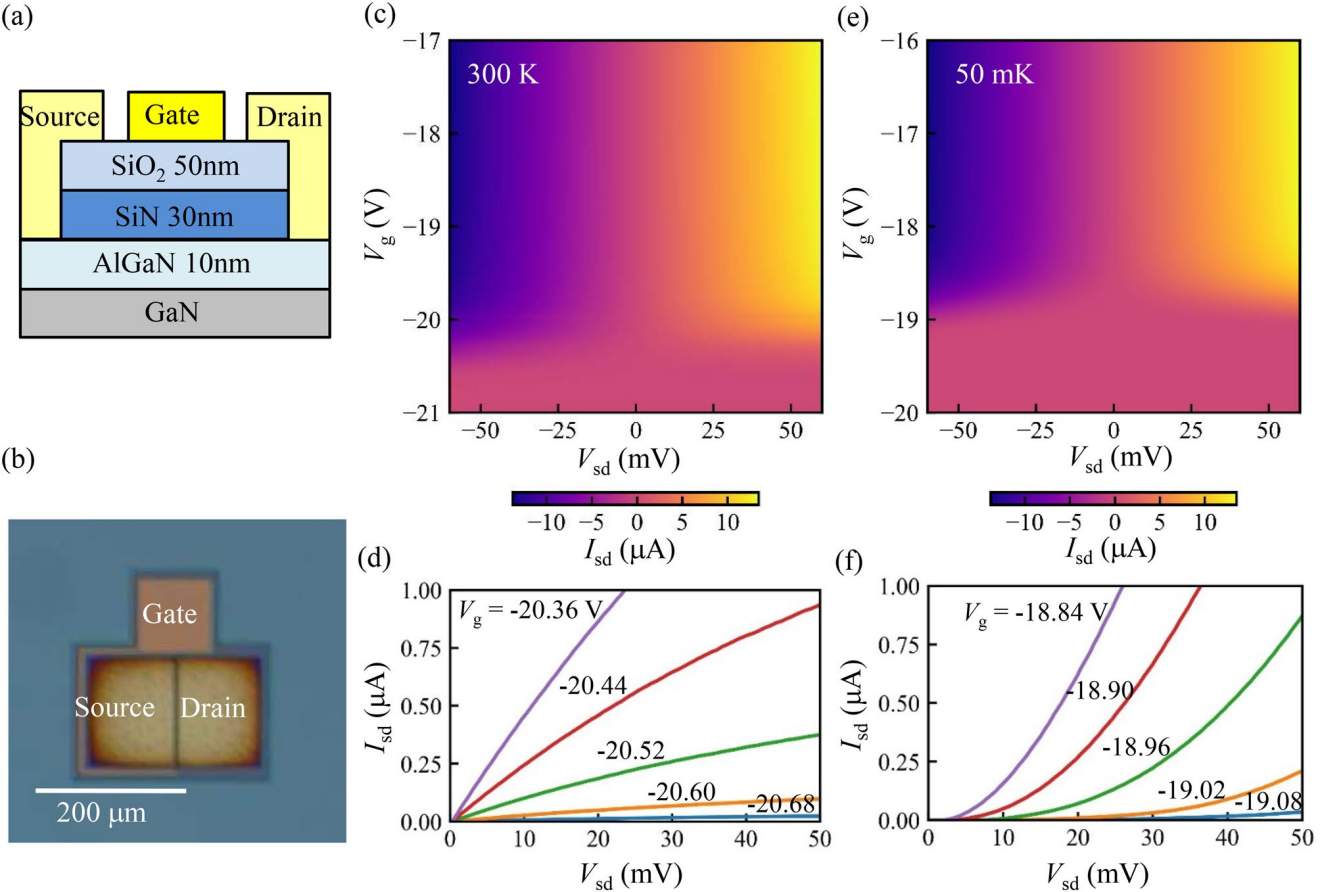
(**a**) Schematic of the layer structure of the device. 2 DEG is formed at the interface between GaN and AlGaN. (**b**) Optical image of the device. The gate electrode with 1.4 µm gate length is placed between the source and the drain. (**c**–**f**) Current trough the GaN/AlGaN FET as a function of the source-drain bias voltage $V_{\text{sd}}$ and the gate voltage $V_{\text{g}}$ at 300 K (**c**,**d**) and 50 mK (**e**,**f**).

Figure 1a shows a schematic of the layer structure of the device. GaN and AlGaN layer is grown on the Si substrate by chemical vapor deposition. At the interface between the GaN and AlGaN layers, 2DEG is formed. The typical values of the electron density and the mobility are $6.7\times 10^{12}\,\hbox {cm}^{-2}$ and $1700~\hbox {cm}^2\hbox {V}^{-1}\hbox {s}^{-1}$. Source and drain contacts are prepared by Ti/Al. A TiN gate electrode is deposited on the insulator of SiN and $\hbox {SiO}_2$. SiN is grown in-situ just after the growth of the GaN/AlGaN. An optical image of the device is Fig. 1b. The gate electrode is placed between the source and the drain contacts. The gate length and the gate width are 1.4 µm and 150 µm, respectively.

The current between the source and the drain contacts $I_{\text{sd}}$ is measured as a function of the applied source-drain bias voltage $V_{\text{sd}}$ and the gate voltage $V_{\text{g}}$. We measure the current through the device at the room temperature 300 K and cryogenic temperature 50 mK. The device is cooled down by a dilution refrigerator.

Figure 1c and d show the measured current through the GaN/AlGaN FET $I_{\text{sd}}$ at the room temperature 300 K. In $V_{\text{g}} > {-} 20.7$ V, the FET channel is opened and the current flows depending on $V_{\text{sd}}$. In the measurement, two 1 kOhm resistors, which is used for low pass filters designed for the cryogenic measurement, are inserted in series to the device and this limits the current in the open condition of the FET. Around $V_{\text{g}} \sim$-20.7 V, the conduction channel is depleted. No current flows in more negatively gated region $V_{\text{g}} <$− 20.7 V.

Figure 1e and f show the measured $I_{\text{sd}}$ at the cryogenic temperature 50 mK. The conduction channel remains at this temperature in $V_{\text{g}} >$− 19.1 V. The depletion of the conduction channel occurs around $V_{\text{g}} \sim$ = − 19.1 V. The pinch-off voltage shifts 1.6 V positively compared to the result at the room temperature. This is induced by the suppression of the thermally induced carriers at the cryogenic temperature. More detail of the sheet resistance and the contact resistance will be revealed by transmission line measurement.

### Formation of quantum dots

**Figure 2:**
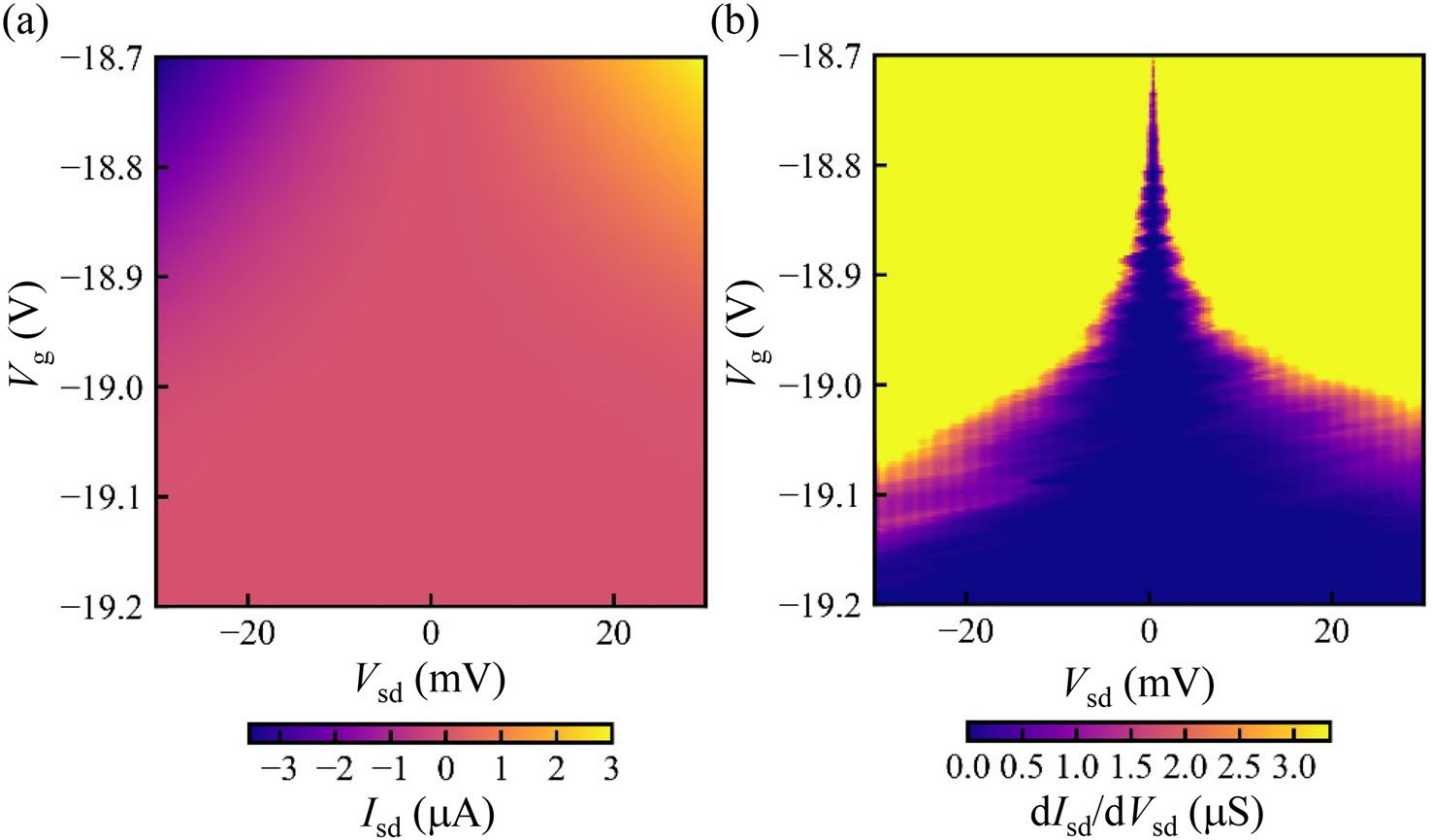
(**a**) Current through the FET as a function of the source-drain bias voltage $V_{\text{sd}}$ and the gate voltage $V_\text{g}$ at 50 mK near the depletion condition of the 2DEG. (**b**) The numerical derivative of the measured current as a function of the source-drain bias voltage $\text{d}I_{\text{sd}}/\text{d}V_{\text{sd}}$. Non-monotonic modulation of the current and Coulomb diamond structures are observed.

Figure 2a shows the current through the FET near the depletion condition of the 2DEG. The current is suppressed around the zero bias and non-linear I–V properties are observed in this region. A numerical derivative of the measured current as a function of the source-drain bias voltage $\text{d}I_{\text{sd}}/\text{d}V_\text{sd}$ is shown in Fig. 2b. The current $I_{\text{sd}}$ is blocked around the zero bias condition $V_{\text{sd}}\sim 0$. The width of the blocked region is modulated by the gate voltage $V_{\text{g}}$ and Coulomb diamonds are observed. The size of the diamonds becomes larger in more negative values of $V_{\text{g}}$ and this reflects that the dot size becomes smaller and the charging energy becomes larger. Note that the faint vertical lines around the outside of the diamonds are the measurement artifact that originates from the output voltages of the source measure unit used in this measurement.

**Figure 3:**
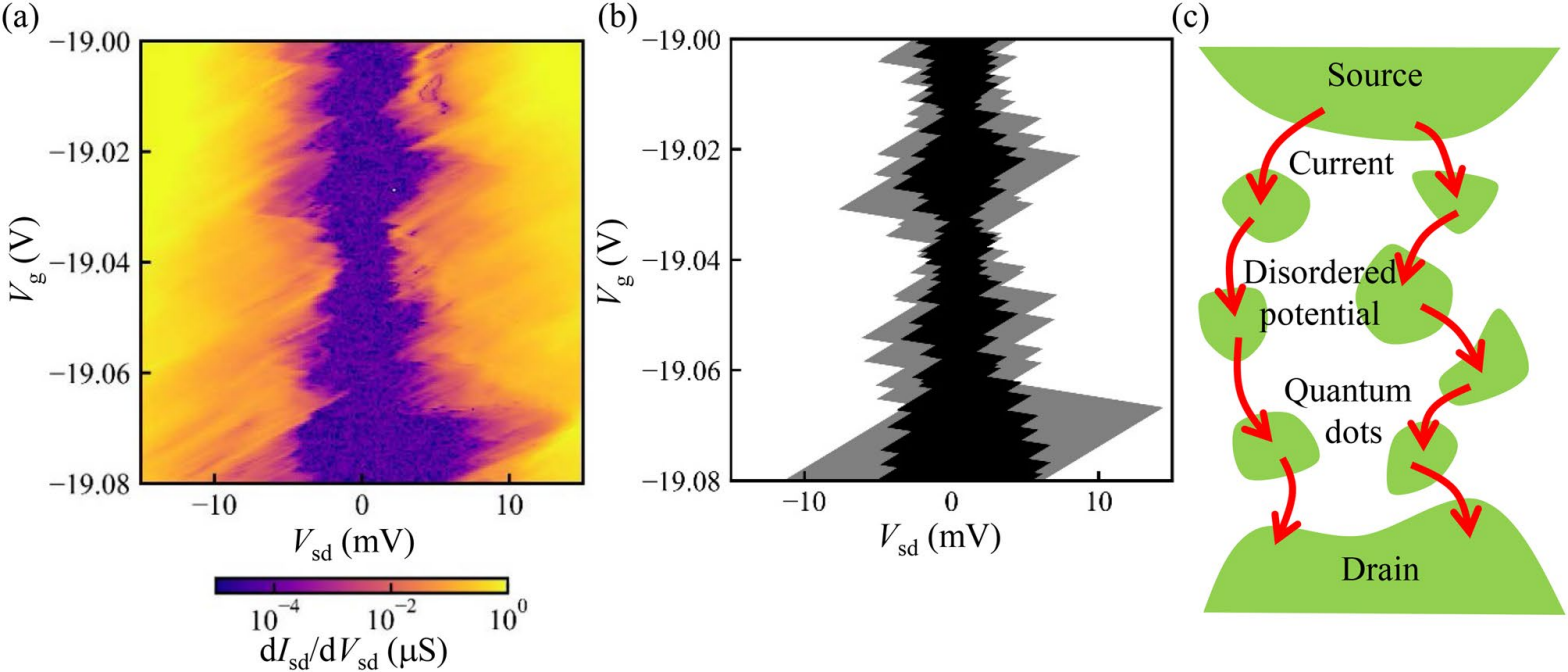
(**a**) Numerical derivative of the current as a function of the source-drain bias voltage. Coulomb diamonds are observed. (**b**,**c**) Schematic of the one possible configuration of the quantum dots (**c**) and the expected Coulomb diamonds (**b**). Electrostatic potential will be disordered by the impurities and defects and the quantum dots are formed at the potential minima. Here we assumed that three and four quantum dots are coupled in series, and these are coupled in parallel. The overlapped Coulomb diamonds show gaps around the zero bias conditions.

Figure 3a shows the closed up image of the Coulomb diamonds. In this small current condition, we use a current preamplifier to measure the current instead of the source measure unit and the measurement artifact like in Fig. 2b is not there. In the Coulomb blodked region, the conductance is smaller than 0.1 nS. The current enhancement by the excited states is also observed as lines outside of the Coulomb diamonds. Quantum dots are formed in the conduction channel of the FET.

The visible lines mostly have the same slope and this indicates that the dot is asymmetrically coupled to the leads: the dot is strongly coupled to one of the leads. The voltage drop by forming the large in-series resistance in the conduction channel is negligible, which can be evaluated by inverting the source and the drain contacts in the measurement^30^. The diamonds are not completely closed at $V_{\text{sd}} = 0$ in Fig. 3a. This shows that multiple quantum dots are formed in this device.

## Discussion

There are no small fine gates or structures to define quantum dots intentionally in this device. The quantum dots will be formed by the disordered potential induced by the impurities or defects near the conduction channel. Near the depletion of the 2DEG, electrons are trapped in the potential minima of the disordered potential. The confinement induces the single-electron charging effect and the size quantization effect. The former produces the Coulomb diamonds. The latter modifies the size of the diamonds and produces the excited state lines.

Figure 3c is a schematic of one possible configuration of the formed quantum dots. Three and four quantum dots are coupled in series, and these are coupled in parallel. The resulting Coulomb diamonds become the overlap of the diamonds of each dot in the case of series connection in a simple approximation^31^. Figure 3b shows the result when we assume the three quantum dots with charging energies $E_{\text{C1}}, E_{\text{C2}}, E_\text{C3} =2.6, 2.3, 3.0$ meV, orbital level spacing $\Delta \epsilon _\text{1} , \Delta \epsilon _{\text{2}}, \Delta \epsilon _{\text{3}} = 0.97{-}11, 0.81{-}4.1, 0.65{-}2.9$ meV and the four quantum dots with charging energies $E_{\text{C4}}, E_{\text{C5}}, E_{\text{C6}}, E_{\text{C7}} =1.4, 1.2, 1.1, 1.3$ meV, orbital level spacing $\Delta \epsilon _{\text{4}}, \Delta \epsilon _{\text{5}}, \Delta \epsilon _{\text{6}}, \Delta \epsilon _{\text{7}} = 0.24{-}3.7, 0.18{-}4.2, 0.18{-}4.8, 0.24{-}4.6$ meV. The gray area indicates the partially blocked region by the three dots and the black area indicates the fully blocked region by the three and four dots. The model capture the main feature of Fig. 3a.

**Figure 4:**
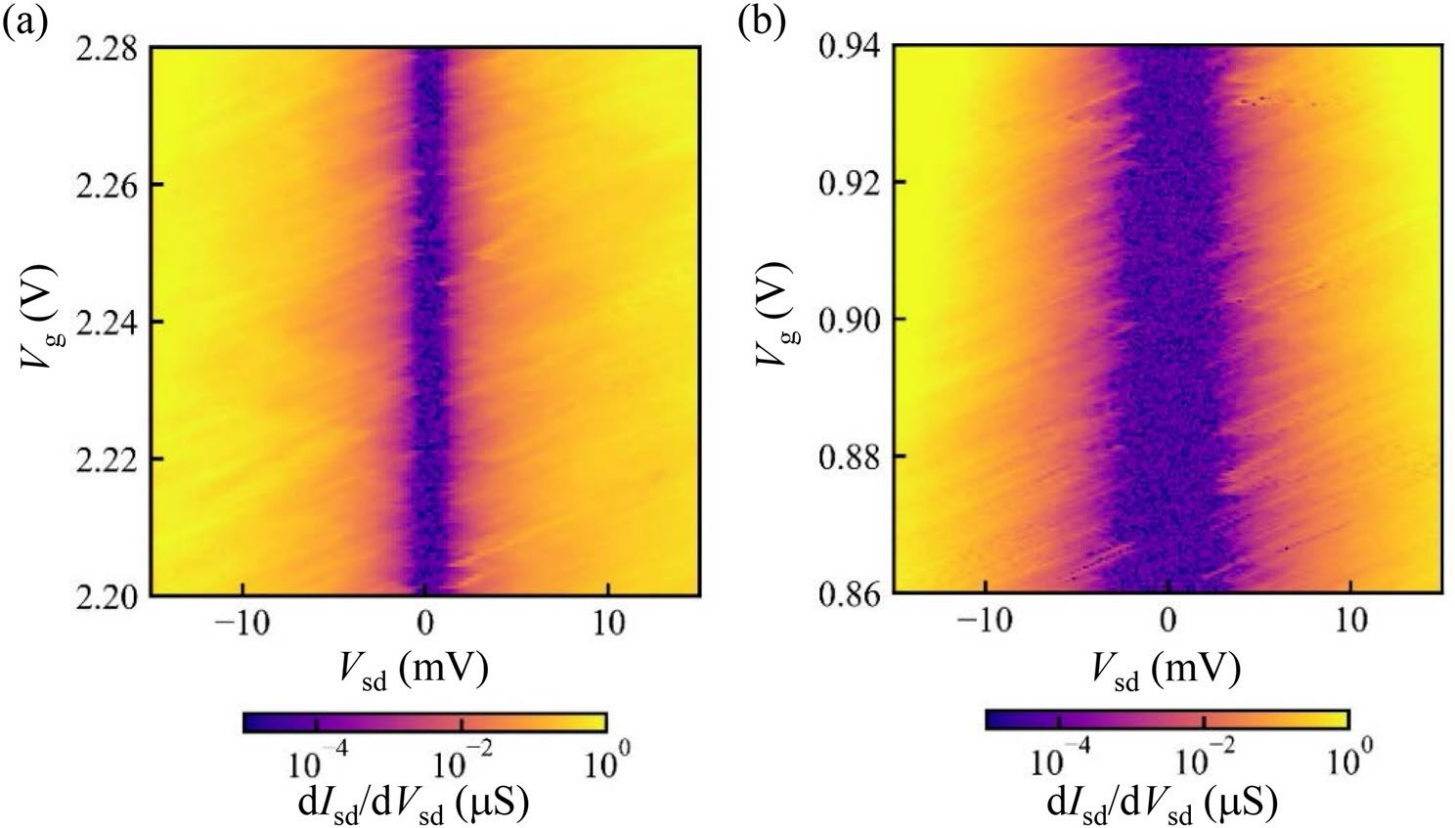
(**a**,**b**) Numerical derivative of the current as a function of the source-drain bias voltage observed in other samples with different insulators $\hbox {SiO}_2$ (**a**) and SiN/$\hbox {SiO}_2$.

To study the growth condition dependence of the quantum dot formation, we measure other samples with different gate insulators and fabrication processes, which induce different disorder densities. In these new samples, the insulator is fabricated after taking out the samples from the growth chamber of GaN/AlGaN and etching processes. The gate length is 0.6 µm. Higher disroder densities are expected compared to the previous sample which has SiN insulators grown in-situ in the same chamber. Figure 4a and b show the results measured in devices with $\hbox {SiO}_2$ and SiN/$\hbox {SiO}_2$ insulators, respectively. Compared to Fig. 3a, more Coulomb diamonds are overlapped and more uniform opening of the gap around the zero bias condition is observed. More quantum dots are formed and coupled in series. The difference between Fig. 4a and b might be caused by variations in oxidization and absorption of molecules outside the chamber. The result in Fig. 4 is consistent with the expectation that the higher disorder density forms more quantum dots in these devices. These support that the origin of the formation of the quantum dots is the disordered potentials around the FET channels.

In conclusion, we measure electron transport in GaN/AlGaN FETs at cryogenic temperature. Quantum dots are formed in the conduction channel near the depletion of the 2DEG. Multiple quantum dots are formed by the disordered potential in the FET. We also measured insulator dependence of the quantum dot formation. These results can be utilized for the development of quantum dot devices like semiconductor quantum bits and nano-probes^32–34^ utilizing GaN/AlGaN and evaluation of the disordered potential in GaN/AlGaN FET channels.

## Supplementary information

Supplementary Information
