# Supplementary material for: Formation of quantum dots in GaN/AlGaN FETs
Source: Sci Rep. 2020 Sep 22;10:15421. doi: 10.1038/s41598-020-72269-z (PMC7508848; doi:10.1038/s41598-020-72269-z)
Supplement: Supplementary file 1 — Supplementary Information [file 41598_2020_72269_MOESM1_ESM.pdf]

Supplemental Material to ‘Formation of quantum dots in GaN/AlGaN FETs’

Tomohiro Otsuka,[1,2,3,4,*] Takaya Abe,[1] Takahito Kitada,[1]
Norikazu Ito,[5] Taketoshi Tanaka,[5] and Ken Nakahara[5]

[1]*Research Institute of Electrical Communication, Tohoku University, 2-1-1 Katahira, Aoba-ku, Sendai 980-8577, Japan*

[2]*Center for Spintronics Research Network, Tohoku University, 2-1-1 Katahira, Aoba-ku, Sendai 980-8577, Japan*

[3]*Center for Science and Innovation in Spintronics, Tohoku University, 2-1-1 Katahira, Aoba-ku, Sendai 980-8577, Japan*

[4]*Center for Emergent Matter Science, RIKEN, 2-1 Hirosawa, Wako, Saitama 351-0198, Japan*

[5]*ROHM Co., Ltd, 21 Saiinnmizosakicho, Ukyo-ku, Kyoto, Kyoto 615-8585, Japan*

(Dated: July 19, 2020)

FET CHARACTERISTICS

Figure S1 shows the FET characteristics of a device with the in-situ SiN insulator and 1.4 μm gate length in the large current condition at the room temperature. The drain-source current $I_{\rm ds}$ is enhanced by opening the conduction channel through the gate voltage $V_{\rm gs}$ and applying the drain-source voltage $V_{\rm ds}$ in Fig. S1(a) and (b). The observed gate leakage current $I_{\rm g}$ in Fig. S1(c) is under the measurement limit.

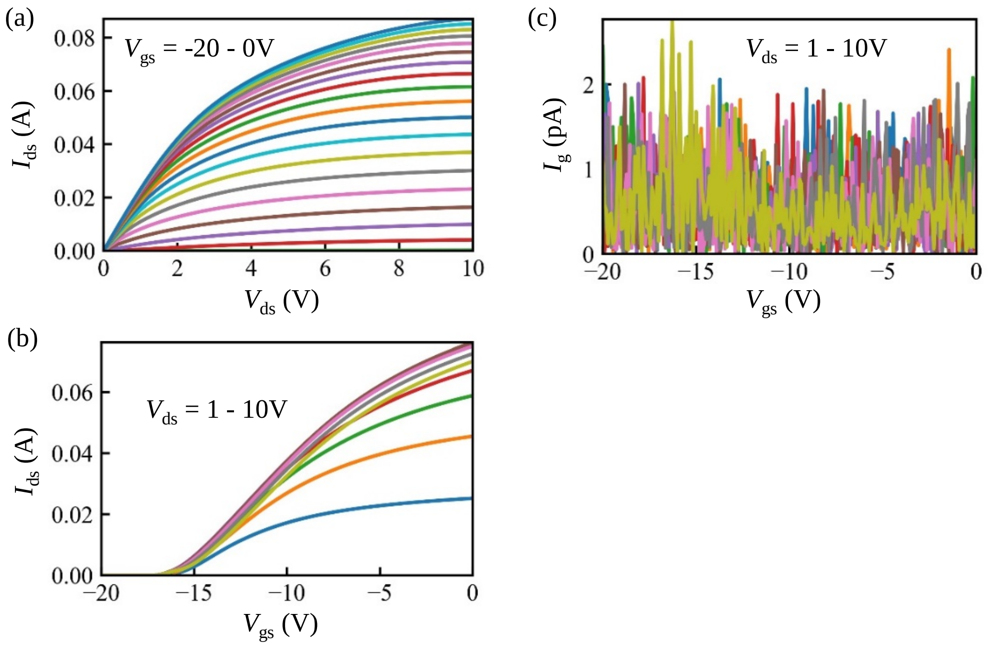

FIG. S1. (a) The drain-source current $I_{\rm ds}$ as a function of the drain-source voltage $V_{\rm ds}$ with changing the gate voltage $V_{\rm gs}$ from -20 to 0 V. (b) $I_{\rm ds}$ as a function of $V_{\rm gs}$ with changing $V_{\rm ds}$ from 1 to 10 V. (c) The gate leakage current $I_{\rm g}$ as a function of $V_{\rm gs}$ with changing $V_{\rm ds}$ from 1 to 10 V.

COULOMB DIAMONDS OBSERVED IN ANOTHER DEVICE

In the main manuscript, we discuss formation of quantum dots in three devices with different insulators. Here we show results observed in another device which is similar to the device in Fig. 1(a) in the main manuscript. The device has the in-situ SiN insulator and the gate length is 1.2 μm.

Figure S2(a) shows the numerical derivative of the measured current as a function of the source-

drain bias voltage $\mathrm{d}I_{\mathrm{sd}}/\mathrm{d}V_{\mathrm{sd}}$. Coulomb diamonds similar to Fig. 2(b) in the main manuscript are observed. Figure S2(b) shows the closed up image of the Coulomb diamonds. The diamonds are not completely closed at $V_{\mathrm{sd}} = 0$ and multiple quantum dots are also formed in this device. These are similar to the results observed in the device in the main manuscript.

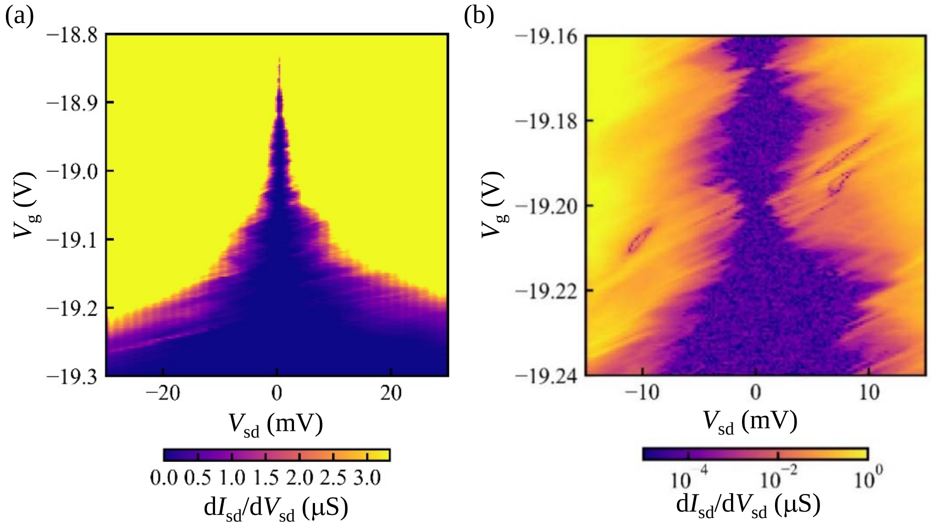

FIG. S2. (a), (b) Coulomb diamonds observed in another device. The numerical derivative of the measured current as a function of the source-drain bias voltage $\mathrm{d}I_{\mathrm{sd}}/\mathrm{d}V_{\mathrm{sd}}$ (a). Close up image of the Coulomb diamonds (b).

* tomohiro.otsuka@riec.tohoku.ac.jp
